# Motivational Processes Associated with Mental Toughness Among College Skiers

**DOI:** 10.3390/bs15050610

**Published:** 2025-05-01

**Authors:** Xinran Wu, Yuhao Cai, Nor Eeza Zainal Abidin, Rafidah Aga Mohd Jaladin

**Affiliations:** 1 Department of Physical Education, Sun Yat-sen University, Guangzhou 510275, China; 2 Department of Physical Education, School of General Education, Guangzhou Institute of Science and Technology, Guangzhou 510540, China; 3 Faculty of Sports and Exercise Science, University of Malaya, Kuala Lumpur 50603, Malaysia; 4 Department of Educational Psychology and Counseling, Faculty of Education, University of Malaya, Kuala Lumpur 50603, Malaysia

**Keywords:** motivational climate, basic psychological needs, motivation, mental toughness, skiing

## Abstract

This study examined the relationships between motivational processes and mental toughness in college skiers based on the self-determination theory (SDT). A total of 332 participants from four skiing items completed four psychological scales assessing motivational factors and mental toughness. The findings revealed that a task-involving climate is positively associated with basic psychological needs, eliciting a positive pathway to autonomous motivation and controlled motivation, thereby positively affecting mental toughness. In contrast, an ego-involving climate is negatively associated with basic psychological needs, eliciting a negative pathway to amotivation, thus negatively affecting mental toughness. The findings highlight the complex interplay between a series of motivational factors and athletes’ mental toughness, underscoring the need to integrate mental-toughness-related factors into the motivational framework.

## 1. Introduction

Skiing is one of the most physically and mentally demanding sports, requiring athletes to perform under extreme conditions, including low temperatures, variable terrain, and high altitudes (Kipp, 2011; Segreti et al., 2024). Success in this discipline depends not only on technical precision and physical conditioning but also on various psychological factors. In particular, mental toughness—a construct defined by the ability to maintain focus, motivation, and performance under stress—is critical for navigating the unique demands of athletes (Jones et al., 2007; Liew et al., 2019; Smith et al., 2013).

Gucciardi et al. (2009) conceptualized mental toughness as a set of values, attitudes, emotions, and cognitions that shape how individuals approach, interpret, and respond to challenging situations in pursuit of their goals. Fourie and Potgieter (2001) further emphasized that motivation is an essential component of mental toughness. Notably, there is a growing body of studies indicating that motivational factors play a pivotal role in fostering and sustaining mental toughness (Crust, 2008; Mahoney et al., 2014a; Reeve, 2024).

The self-determination theory (SDT), an important theory for understanding motivation, highlights how intrinsic motivation, driven by the satisfaction of autonomy, competence, and relatedness, can enhance resilience and persistence in high-pressure contexts. Conversely, extrinsic motivation, shaped by external rewards or pressures, may contribute to performance but often lacks the long-term benefits associated with intrinsic drives (Deci & Ryan, 2012). Understanding the motivational factors that underpin mental toughness is essential for developing effective interventions and optimizing athlete performance.

In the field of sport psychology, extensive research has explored how motivational factors and mental toughness interact across various sports. For instance, studies in golf (Schaefer et al., 2016), tennis (Gucciardi et al., 2015), running (Mahoney et al., 2014b), and team sports such as basketball (Asamoah, 2013) and football (Butt et al., 2010) have shown that athletes with a higher level of motivational-related factors report greater mental toughness and stress tolerance. These findings suggest that motivation not only drives athletic engagement and goal persistence but also serves as a psychological buffer in high-pressure situations and thus contributes to the development of mental toughness.

Despite growing recognition of the interplay between motivational factors and mental toughness, there is limited research aiming to understand mental toughness in the context of overall motivational processes, especially within the context of winter sports. Winter sports encompass a diverse range of athletic items, including team sports like ice hockey and curling, as well as individual events such as skiing and snowboarding. While these items differ in their technical and tactical demands, they share unique challenges for athletes (Sandbakk & Holmberg, 2017; Willmott & Collins, 2015). From a biomechanical and physiological perspective, these challenges include performing a variety of difficult technical maneuvers on snow and ice using specialized equipment such as skis, skates, and sledges, as well as enduring environmental stresses such as extreme cold and oxygen deprivation at high altitudes (Ma et al., 2023). In such demanding contexts, mental toughness becomes a crucial factor for success.

Understanding the factors that influence mental toughness is crucial for optimizing athlete health, training strategies, and performance outcomes. Drawing on theories related to motivation and mental toughness, this study examines the motivational processes associated with mental toughness in college skiers. The objective of this research is to provide motivational-process-based strategies for enhancing mental toughness, ultimately contributing to improved performance and well-being in skiers, with broader implications for the winter sports community.

## 2. Mental Toughness in Sport

Mental toughness is a psychological construct widely regarded as essential for athletic success. It encompasses an athlete’s ability to consistently perform at their best under pressure, maintain resilience in the face of setbacks, and sustain focus and motivation over extended periods (Clough et al., 2002; Sheard et al., 2009). Early conceptualizations of mental toughness emphasized its multidimensional nature, including components such as confidence, resilience, focus, and emotional regulation (Jones et al., 2007). Clough et al. (2002) introduced the 4Cs model, defining mental toughness through control, commitment, challenge, and confidence. This model has since guided much of the research in the field, offering a structured approach to operationalizing mental toughness in both research and practice.

Sheard et al. (2009) developed the sports mental toughness questionnaire (SMTQ), a self-report instrument for assessing sports mental toughness. It contains fourteen items and three subscales, including confidence, constancy, and control. More recent perspectives advocate for a dynamic understanding of mental toughness, highlighting its situational and developmental aspects. Gucciardi et al. proposed a process-oriented framework, emphasizing the role of context, individual differences, and environmental factors in shaping mental toughness (Gucciardi et al., 2009). This perspective underscores the interaction between innate predispositions and external influences, such as coaching style, peer interactions, and competitive experiences.

The development of mental toughness is influenced by a combination of innate traits and environmental conditions. Studies suggest that exposure to challenging yet supportive environments fosters resilience and adaptability, key components of mental toughness (Bell et al., 2013). Positive coaching practices, peer support, and mastery-oriented climates have been shown to enhance athletes’ mental toughness by promoting self-efficacy and adaptive coping strategies (Crust & Clough, 2011). Conversely, overly critical feedback or excessively competitive environments can hinder the development of mental toughness, leading to negative psychological outcomes such as burnout or anxiety (Gucciardi et al., 2016). These findings highlight the importance of creating balanced training environments that challenge athletes while providing emotional support and constructive feedback.

Notably, mental toughness is strongly associated with performance consistency, particularly in high-pressure situations. Athletes with higher levels of mental toughness demonstrate greater resilience to stress, superior focus during competition, and enhanced ability to recover from errors (Cowden, 2017). These attributes enable them to maintain performance levels across diverse competitive settings, often distinguishing athletes from their peers. On the other hand, mental toughness contributes to team dynamics. Research indicates that mentally tough athletes often serve as role models, fostering a culture of resilience and determination within their teams (Mahoney et al., 2014a). This collective benefit underscores the value of integrating mental toughness training into training development programs.

Mental toughness is one of the critical determinants of athletic success, characterized by its multifaceted, dynamic nature and its significant impact on performance, all of which are integral to achieving success in competitive sports. Conceptual advancements and empirical findings underscore the importance of fostering mental toughness through balanced and supportive environments.

## 3. Theoretical Integration of SDT and HMIEM

Mental toughness is a key psychological characteristic closely linked to outcomes and success in elite sports, making it essential to understand the motivational mechanisms that underpin its development (Crust, 2007; Mahoney et al., 2014b). The self-determination theory (SDT) and the hierarchical model of intrinsic and extrinsic motivation (HMIEM) provide valuable frameworks for exploring the complex dynamics of motivation, including the interaction of intrinsic drives, extrinsic influences, and psychological well-being. Integrating these theories with research on athletes’ mental toughness offers a more comprehensive understanding of the factors influencing their behaviors, performance, and overall experiences.

Understanding athletes’ motivation is fundamental to examining the development of mental toughness. The SDT serves as a comprehensive framework for exploring the interplay between extrinsic and intrinsic motivation, emphasizing the significance of fulfilling each dimension of basic psychological needs: autonomy, competence, and relatedness (Deci & Ryan, 2013). People are more likely to develop intrinsic motivation when these demands are satisfied, which has been associated with enhanced persistence, psychological well-being, and optimal performance (Ryan & Deci, 2000).

The HMIEM extends the SDT by advocating for a holistic exploration of motivational outcomes, encompassing affective, behavior, and cognitive dimensions (Vallerand, 1997). It is conceptually framed as a schematic representation of motivational dynamics, highlighting the association among theoretical constructs and serving as a foundational framework for quantitative research (Sheehan et al., 2018; Stenling et al., 2015). This approach provides a holistic understanding of the motivational process by examining how various levels of motivation interact to shape emotional responses, cognitive appraisals, and behavioral patterns across different contexts and situations. Specifically, it provides a compelling framework for understanding motivational processes, consisting of four key stages, social factors → basic psychological needs → motivation → consequences, and organizes motivation into three levels: global, contextual, and situational. This study primarily examines this motivational sequence at the contextual level, particularly in the sports context. The HMIEM highlights how motivation is influenced by social environments, such as coach–athlete relationships, and how it impacts outcomes ranging from engagement to psychological well-being (Vallerand, 1997).

Integrating the SDT and HMIEM provides a nuanced understanding of the motivational processes underlying mental toughness in athletes. Research indicates that motivational climates fostering autonomy support and competence reinforcement are positively linked to higher levels of intrinsic motivation and resilience (Ntoumanis & Mallett, 2014). Conversely, environments dominated by controlling behaviors and extrinsic rewards can undermine intrinsic motivation, potentially leading to mental problems (Mahoney et al., 2014b; Vallerand, 1997). By synthesizing these theoretical perspectives, this review highlights the critical role of motivation in shaping athletes’ mental toughness and performance outcomes. Further exploration of the SDT and HMIEM within sports psychology offers valuable insights into designing interventions to enhance psychological resilience and long-term athlete well-being.

## 4. Motivational Factors and Mental Toughness

Mental toughness is a critical psychological construct in sports, enabling athletes to navigate challenges, maintain focus, and achieve consistent performance under pressure. Over the years, sports psychologists and researchers have explored the intricate associations among various motivational factors and emphasized their impact on athletes’ psychological resilience and sustained success (Gucciardi et al., 2009).

The achievement goal theory (AGT) provides a foundational framework for understanding motivation in sports, particularly through the concept of motivational climate (Harwood et al., 2008). A task-involving climate, characterized by an emphasis on effort, improvement, and mastery, has been shown to promote confidence, perseverance, and adaptive responses to setbacks—all key attributes of mental toughness (Beck et al., 2017; Nicholls et al., 2016; Olvera, 2020). Conversely, an ego-involving climate, which prioritizes outperforming others, can lead to increased anxiety, fear of failure, and decreased resilience (Duda & Balaguer, 2007; Ramirez & Delariarte, 2024). These studies suggest that athletes in a task-involving environment exhibit more positive psychological states than athletes in an ego-involving climate (Harwood et al., 2015).

The SDT further elucidates the link between motivation and mental toughness by focusing on the fulfillment of basic psychological needs: autonomy, competence, and relatedness (Deci & Ryan, 2000). Autonomy, or the sense of volitional control, empowers athletes to approach challenges with intrinsic motivation, fostering persistence and self-regulation. Competence, derived from skill mastery and performance improvement, enhances self-efficacy and problem-solving abilities in high-pressure situations. Relatedness, rooted in supportive relationships, contributes to a sense of belonging and emotional stability, which underpin mental toughness. Research has shown that the satisfaction of these needs positively correlates with athletes’ ability to manage stress and maintain performance consistency (Vallerand, 2007).

Individual motivation, encompassing intrinsic and extrinsic dimensions, also influences mental toughness. Intrinsically motivated athletes, driven by genuine passion and enjoyment, tend to exhibit greater psychological resilience and sustained effort over time (León-Guereño et al., 2020). Extrinsic motivation, when aligned with personal goals, can complement intrinsic drives, providing additional reinforcement (Morris et al., 2022). Conversely, amotivation—marked by a lack of purpose or disengagement—undermines mental toughness, heightening vulnerability to stress and performance decline. This often arises from an inability to perceive the activity’s value or a sense of inadequacy in one’s ability to perform it (Jackson, 2016).

Moreover, autonomous motivation, as defined within the SDT, encompasses identified regulation, integrated regulation, and intrinsic regulation, arising from internal and external sources. Individuals with higher autonomy in their motivation are primarily driven by the value they assign to a behavior, such as finding it personally interesting or enjoyable. This autonomy fosters persistence, satisfaction, and enhanced overall well-being (Ryan & Deci, 2000). Controlled motivation includes introjected and external regulation, which are primarily influenced by external factors such as rewards, punishments, or social pressures. According to the SDT, autonomous motivation is associated with a range of positive outcomes, including healthier behaviors, sustained commitment, and improved performance. These benefits underscore the importance of fostering autonomy to promote long-term engagement and well-being (Deci & Ryan, 2008).

In summary, motivational factors influence the development and maintenance of mental toughness in athletes. Frameworks such as the SDT and AGT provide valuable insights into how task-involving climates and the fulfillment of basic psychological needs contribute to resilience and performance under pressure. The HMIEM provides a theoretical model that encompasses the processes of motivational climate, basic psychological needs, motivation, and mental consequences. This research seeks to deepen our understanding of these relationships and provide practical recommendations to coaches and athletes for fostering mental toughness through effective motivational strategies.

## 5. The Present Research

Building on the theoretical and empirical foundations discussed earlier, this study adopted a quantitative cross-sectional survey design. Data were collected using an ordinal scale research instrument, designed to assess variables sequentially. The primary objective was to test the four stages of the motivational sequence within the sports context. The HMIEM was employed to incorporate mental toughness into the motivational sequence at the contextual level, which was conceptualized as follows: motivational climate → basic psychological needs → sports motivation → mental toughness. This study proposed the following hypotheses:

**Hypothesis** **1.** *An ego-involving climate is negatively related to basic psychological needs, while a task-involving climate is positively related to basic psychological needs.*

**Hypothesis** **2.** *Basic psychological needs are negatively related to amotivation and positively related to autonomous motivation, while they have a positive association or no association with controlled motivation.*

**Hypothesis** **3.** *Autonomous motivation is positively related to mental toughness, whereas amotivation is negatively related to mental toughness. Controlled motivation is positively or not related to mental toughness.*

## 6. Method

### 6.1. Participants

The survey study included 332 college skiers (59.0% male, 41.0% female) who were recruited via convenience sampling. The demographic information of subjects is shown in Table 1. The participants, representing four skiing disciplines, were recruited from three sports universities in northeast China: alpine skiing (*n* = 179, 53.9%), snowboarding (*n* = 52, 15.7%), freestyling (*n* = 65, 19.6%), and cross-country skiing (*n* = 36, 10.8%). Among participants aged 18 to 25, 84.6% of them had more than five years of training experience. Moreover, 3.9% of participants achieved the international master level (the highest competitive level), 10.2% of them reached the national master level (the second competitive level), 39.5% of them reached the national first level (the third competitive level), and 46.4% of them reached the national second level (the fourth competitive level). In total, 42.5% of them trained 4–5 times per week, and 44.6% of them trained 2–3 times per week, with an average training time for all participants of 120 min per session.

### 6.2. Measures

This study utilized a comprehensive survey comprising 85 items drawn from four validated instruments to assess motivational climates, basic psychological needs, motivation, and mental toughness. The survey was designed to collect a comprehensive range of data to provide a nuanced understanding of the subjects’ perceptions and experiences within the sporting context. The details of each original version of the instrument are summarized below.

#### 6.2.1. Perceived Motivational Climate in Sport Questionnaire II (PMCSQ-II)

The PMCSQ-II, a widely used instrument for evaluating motivational climates in sports, consists of 33 items measuring athletes’ perceptions of task-involving and ego-involving climates. Responses were recorded on a 5-point Likert scale ranging from 1 (Strongly disagree) to 5 (Strongly agree). The instrument demonstrated high reliability in a study by Newton et al. (2000), with Cronbach’s α coefficients of 0.87 for the ego climate subscale and 0.88 for the task climate subscale.

#### 6.2.2. Basic Need Satisfaction in Sport Scale (BNSSS)

The BNSSS is a 20-item scale designed to assess athletes’ experiences of competence, autonomy, and relatedness during sports participation. Participants rated their basic psychological need satisfaction using a 7-point Likert scale ranging from 1 (Not at all true) to 7 (Very true). The BNSSS showed acceptable reliability, with Cronbach’s α coefficients for its five subscales ranging from 0.61 to 0.82 (Ng et al., 2011).

#### 6.2.3. Sport Motivation Scale II (SMS-II)

The SMS-II was used to assess the athletes’ motivation through 18 items. Participants responded to questions about their reasons for engaging in their respective sports. Responses were rated on a 7-point Likert scale, ranging from 1 (Not at all true) to 7 (Very true). It is a validated measure of sports motivation, with Cronbach’s α coefficients for its six subscales ranging from 0.73 to 0.86, indicating strong reliability (Pelletier et al., 2013). Autonomous motivation was assessed by averaging the items related to intrinsic motivation, integration, and identification, while controlled motivation was determined by averaging the items associated with introjection and extrinsic regulation. Amotivation was calculated by averaging no-regulation-related items.

#### 6.2.4. Sports Mental Toughness Questionnaire (SMTQ)

The SMTQ is a self-report instrument designed to assess mental toughness in sports. It comprises 14 items rated on a 4-point Likert scale ranging from 1 (Not at all true) to 4 (Very true). The SMTQ includes three subscales: confidence (6 items), constancy (4 items), and control (4 items). The instrument demonstrated acceptable reliability, with Cronbach’s α coefficients of >0.70 for each subscale (Sheard et al., 2009).

### 6.3. Translation Procedures

The survey instruments were originally developed in English and required adaptation for Chinese-speaking participants. Before data collection, the questionnaires were subjected to a rigorous validation process to ensure linguistic and contextual accuracy. Although the PMCSQ-II (Cai, 2016), BNSSS (Ng, 2008), SMS-II (Li et al., 2018), and SMTQ (J. Zheng, 2014) had been previously translated for Chinese-speaking samples, they were included in the validation process to confirm their suitability for this study. To ensure equivalence and accuracy, the translated questionnaires were reviewed by three experts in sports psychology. Their feedback affirmed that the Chinese versions were appropriate for use in the survey.

### 6.4. Data Collection

Data collection began after receiving approval from the Research Ethics Committee (REC) and the acquisition of permissions from three universities in northeast China. Prior to distributing the questionnaires, researchers secured the cooperation of team coaches and institutional leaders by email invitations. The informed consent form was sent by email to the coaches and printed in paper form, and the subjects filled out the paper version of the informed consent form. During the investigation, subjects self-administered the questionnaires and completed the paper-based survey under the guidance of a research assistant, ensuring their coaches were not present during the survey process. To guarantee clarity and uniformity, standardized written and verbal instructions were provided before participants began the survey. These instructions were designed to improve the participants’ comprehension of each question and assist them in managing their time effectively. The survey, consisting of 85 questions, took approximately 30 minutes to complete.

### 6.5. Data Analysis

The data analysis was conducted in the following steps:

Step 1: Preliminary analyses were performed using SPSS Statistics 26.0 to screen for missing and out-of-range values, assess normality, and evaluate reliability. Internal consistency was measured using Cronbach’s α, with a cut-off score of 0.70 deemed acceptable (Nunnally, 1978).

Step 2: Pearson’s correlation coefficient was employed to examine relationships among variables, with values interpreted as small (0.10), moderate (0.30), and large (0.50) (Cohen, 2013). Descriptive statistics were calculated to summarize the participants’ scores on the measuring scales.

Step 3: SEM was conducted using AMOS 26.0 to evaluate and specify a conceptual model describing the associations between mental toughness and a set of motivational variables. Model fit was assessed using the following criteria: TLI, CFI, and GFI values > 0.90 were considered indicative of a good fit (Kline, 2005). RMSEA and SRMR values < 0.08 denoted acceptable fit (Hu & Bentler, 1999). CMIN/DF values < 5 were considered reasonable (Marsh & Hocevar, 1985). Standardized regression coefficients were used to quantify relationships between variables, with effect sizes classified as small (0.10), moderate (0.30), and large (0.50) (Cohen, 1992).

## 7. Results

### 7.1. Preliminary Analysis

A small subset of participants (n = 8) completed questionnaires containing minor amounts of missing data. To address this, missing values for each case were replaced with the mean of the available items within the corresponding subscale (Graham et al., 2003). The dataset underwent rigorous examination to identify potential univariate and multivariate outliers. Following Tabachnick et al.’s (2013) guidelines, data were deemed normal if the standardized z-scores fell within the range of ±3.29. No responses exceeded this threshold, confirming the absence of univariate outliers. However, Mardia’s normalized coefficient (21.42) indicated a departure from multivariate normality. Given the sample size and the observed deviation, a bootstrapping procedure with 5000 iterations was implemented to enhance the stability of parameter estimates in multivariate analyses (Hayes, 2009).

Cronbach’s α coefficients were computed to assess the internal consistency reliability of the measuring scales (see Table 2), with values ranging from 0.81 to 0.93. Specifically, the coefficients were as follows: task-involving climate (*α* = 0.93) and ego-involving climate (*α* = 0.91); competence (*α* = 0.90), autonomy (*α* = 0.87), and relatedness (*α* = 0.81); autonomous motivation (*α = 0.90*), controlled motivation (*α* = 0.83), and amotivation (*α* = 0.85); confidence (*α* = 0.86), constancy (*α* = 0.85), and control (*α* = 0.85); all values were above the threshold of 0.70 (Nunnally, 1978). The results indicated that all subscales had acceptable internal consistency.

### 7.2. Descriptive Statistics

Several key findings can be derived from the means and standard deviations presented in Table 2. First, the motivational climate measures revealed a higher mean for the task-involving climate (*M* = 4.31, *SD* = 0.70) compared to the ego-involving climate (*M* = 3.08, *SD* = 0.98). Second, participants reported high levels across each dimension of basic psychological needs: competence (*M* = 5.34, *SD* = 1.23), autonomy (*M* = 5.10, *SD* = 1.10), and relatedness (*M* = 5.82, *SD* = 1.11). Third, the participants exhibited low levels of amotivation (*M* = 2.75, *SD* = 1.33) and high levels of autonomous motivation (*M* = 5.55, *SD* = 1.01). Finally, the scores were high across the three dimensions of mental toughness, including confidence (*M* = 3.78, *SD* = 0.53), constancy (*M* = 3.75, *SD* = 0.56), and control (*M* = 3.73, *SD* = 0.53).

Additionally, Pearson correlations between mental toughness and various motivational factors (see Table 2) revealed several statistically significant relationships. There were significantly more positive correlations than negative correlations. Notably, the task-involving climate, autonomous motivation, controlled motivation, and each dimension of the basic psychological needs were positively associated with mental toughness. Among these different variables, the strongest significant correlation was observed between autonomy and autonomous motivation (*r* = 0.46, *p* < 0.01). In contrast, the weakest significant correlation was between constancy and the task-involving climate (*r* = 0.10, *p* < 0.05).

### 7.3. Structural Equation Modeling

Consistent with the formulated hypotheses, the model integrates five manifest variables—ego-involving climate, task-involving climate, amotivation, autonomous motivation, controlled motivation—and two latent variables: mental toughness and basic psychological needs. Specifically, the variable of mental toughness used three subscales (confidence, constancy, and control) as measurements, and the variable of basic psychological needs was created using three subscales (autonomy, competence, and relatedness) as measurements.

**Figure 1:**
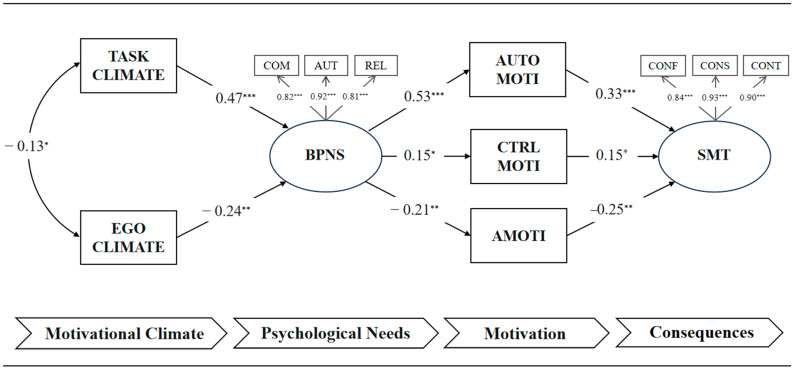
Standardized path coefficients from the SDT structural models. Note: BPNS = basic psychological needs satisfaction; COM = competence; AUT = autonomy; REL = relatedness; AUTO MOTI = autonomous motivation; CTRL MOTI = controlled motivation; AMOTI = amotivation; SMT = sports mental toughness; CONF = confidence; CONS = constancy; CONT = control; * *p* < 0.05, ** *p* < 0.01, *** *p* < 0.001.

The self-determination theory (SDT)-based model, which examines the data on mental toughness and various motivational factors, demonstrated an acceptable fit (χ^2^ = 134, df = 40, *p* < 0.001, GFI = 0.94, CFI = 0.95, TLI = 0.94, SRMR = 0.06, RMSEA = 0.08, 90% CI = 0.06 to 0.09; see Figure 1). To ensure the stability of parameter estimates within the structural model, bootstrapping with 5000 iterations was performed. The results, presented in Table 3, indicated high stability of the parameter estimates. The findings supported this study’s hypotheses, revealing the following relationships:

(a) A strong positive correlation (*β* = 0.47) was observed between the task-involving climate and basic psychological needs, whereas a small-to-moderate negative correlation (*β* = −0.24) was identified between the ego-involving climate and basic psychological needs.

(b) A strong positive correlation (*β* = 0.53) emerged between basic psychological needs and autonomous motivation, whereas a small-to-moderate negative correlation (*β* = −0.21) was observed between basic psychological needs and amotivation. Moreover, a small positive correlation (*β* = 0.15) was observed between basic psychological needs and controlled motivation.

(c) A moderate positive correlation (*β* = 0.33) was found between autonomous motivation and mental toughness, whereas a small-to-moderate negative correlation (*β* = −0.25) was identified between amotivation and mental toughness. Moreover, a small positive correlation (*β* = 0.15) was found between controlled motivation and mental toughness.

### 7.4. Assessment of Mediation

All indirect effects were statistically significant (*p* < 0.05) with the exception of the effects of the ego-involving climate on sports mental toughness via basic psychological needs satisfaction and controlled motivation. The indirect effects are displayed in Table 4. The analyses indicated that the task-involving climate and sports mental toughness associations were mediated by basic psychological need satisfaction and three types of motivation. The associations between ego-involving climate and sports mental toughness are mediated by basic psychological need satisfaction, autonomous motivation, and amotivation.

## 8. Discussion

This study extends previous motivation research grounded in the SDT by evaluating a model that links various motivational factors to mental toughness in the HMIEM. The findings support the proposed model, demonstrating that mental toughness is a key component of the motivational processes. Additionally, the results support the hypothesized correlations within the motivational model. Specifically, college skiers exhibited positive motivational patterns, including high levels of autonomous motivation, task-involving climate, and fulfillment of basic psychological needs, all of which were associated with higher mental toughness. The present research contributes to the literature by exploring the relationship between traditional elements and mental toughness in the HMIEM. Furthermore, the findings offer practical insights that may help address real-life challenges faced by college athletes, including underperformance and mental health issues.

### 8.1. Motivational Climate and Basic Psychological Needs

The results offer compelling evidence in favor of the hypothesized model. Specifically, the findings reveal that the athletes’ perceptions of a task-involving climate are positively associated with their basic psychological need satisfaction, aligning with earlier studies (Chen et al., 2020; Hodge & Gucciardi, 2015; S. Zheng et al., 2023). In addition to emphasizing skill development, coaching that fosters a task-involving environment also helps athletes build strong, sustaining connections. Over the long term, such a climate instills a sense of purpose, cultivates self-beliefs, and values autonomy, leading to enhanced need satisfaction.

Conversely, an ego-involving climate negatively impacts basic psychological need satisfaction, echoing findings from prior studies (Gråstén & Watt, 2017). Such a climate prioritizes ego-driven outcomes and individual achievements over athletes’ well-being and holistic growth. It reduces the importance of fostering healthy relationships, nurturing positive beliefs, and respecting autonomy, which in turn undermines athletes’ psychological needs.

In competitive environments where intra-team comparisons dominate and recognition is reserved for top performers, athletes’ sense of connection and belonging within the team may be compromised. The current study indicates that an ego-involving climate may hinder need satisfaction, as these athletes benefit from being accepted, valued, and nurtured by those whose opinions matter most. Nevertheless, the findings contrast with those of García-González et al. (2019), who found no significant relationship between the ego-involving climate and basic needs satisfaction. This discrepancy may be due to differences in sample populations or the possibility that the ego-involving climate did not significantly affect some participants. It is plausible that moderating variables, such as perceived ability, may influence the relationship between basic psychological needs and ego-involving climates.

Overall, the results underscore the critical role of motivational climates in sports, particularly in shaping athletes’ basic psychological needs. They highlight the importance of cultivating a task-involving climate that promotes personal growth, cooperation, and supportive relationships while discouraging ego-involving behaviors, including excessive comparisons and reliance on external validation. Such strategies are essential for enhancing athletes’ psychological well-being and overall performance.

### 8.2. Basic Psychological Needs and Motivation

The results showed a positive association between basic psychological needs and autonomous motivation, as well as a negative relationship to amotivation. The findings provide useful insights into motivational elements in winter sports, emphasizing the crucial role of meeting basic psychological needs in promoting athletes’ motivation. This supports prior research across diverse domains, including education and sports, where satisfying basic psychological needs consistently enhances autonomous motivation.

The self-determination theory (SDT) posits that individual’s motivation require autonomy, competence, and relatedness to meet basic psychological needs. Our findings align with this framework, showing that these needs are pivotal in shaping motivational outcomes, allowing athletes to experience greater intrinsic motivation, engagement, and active participation in sports. This aligns with existing research across various domains, such as education and sports, where each dimension of basic psychological needs positively influences autonomous motivation (Robazza et al., 2023; Ryan, 2017; Tang et al., 2021). Moreover, basic psychological needs have a relatively small positive impact on controlled motivation, while others have reported a negative relationship between basic psychological needs and controlled motivation (Bartholomew et al., 2018). Conversely, the observed negative relationship between basic psychological needs and amotivation aligns with prior studies (Chevrier & Lannegrand, 2022; Leo et al., 2022; Zamarripa et al., 2021). This suggests that unmet or frustrated psychological needs increase the likelihood of demotivation or indifference toward sports. Amotivation, characterized by a lack of intention or drive to engage, often stems from feelings of disconnection, incompetence, or external control. Such encounters might cause disengagement and decreased motivation (Guay et al., 2003).

The findings offer practical insights for winter sports programs and coaching strategies aimed at enhancing athlete motivation and performance. By meeting athletes’ basic psychological needs, coaches can cultivate a supportive atmosphere that enhances autonomous motivation while reducing amotivation. Specifically, providing athletes with opportunities for autonomy—such as involving them in decisions about training and competition schedules—can enhance their sense of control. Competence can be bolstered through tailored skill development programs and constructive feedback. Additionally, promoting relatedness by fostering social support and belonging within the team can strengthen positive associations and team cohesion.

In summary, meeting athletes’ basic psychological needs is essential for increasing autonomous motivation and mitigating amotivation. Coaches and practitioners can use these insights to design environments and interventions that promote athletes’ psychological well-being, driving greater motivation, engagement, and performance in sports.

### 8.3. Motivation and Mental Toughness

The present research provides important insights into the function of motivation in shaping and enhancing mental toughness among college skiers. The results reveal distinct correlations between motivational processes and mental toughness, underscoring the significance of understanding motivational factors in cultivating mental toughness in athletes.

The findings indicated that autonomous motivation, characterized by the enjoyment and intrinsic drive of the activity, was positively correlated with mental toughness. This suggests that winter athletes with strong autonomous motivation may have strong mental toughness, including confidence, constancy, and control. These findings are consistent with existing research that identifies autonomous motivation as a positive contributor to mental toughness (Scharneck, 2017). Athletes who experience authentic satisfaction and pleasure in their sport may be better prepared to handle the stresses and inherent challenges of competition, thus improving their mental toughness. Moreover, a small positive correlation was found between controlled motivation and mental toughness, which is consistent with a previous study (Cowden et al., 2021).

Conversely, our results also revealed that amotivation, characterized by a lack of motivation or perceived irrelevance to the activity, was negatively correlated with mental toughness. This aligns with prior studies (Cowden et al., 2019), suggesting that athletes who feel unmotivated or disconnected from their sport are more likely to be characterized by a lack of mental toughness. It might be difficult for unmotivated athletes to find meaning or purpose in their physical endeavors, making them more susceptible to emotional exhaustion and reduced mental toughness. These findings highlight the detrimental effects of amotivation on athletic performance and mental health (Vallerand et al., 1992).

Notably, the participants report high levels of mental toughness. The result may also be attributed to the support from coaches and university leadership. These universities provided mental health counseling services both on campus and at remote ski resorts, which likely contributed to enhancing mental toughness among athletes.

These findings underscore the importance of cultivating autonomous motivation and solving the issues of amotivation in order to enhance the mental toughness and well-being of college skiers. Related practitioners should implement strategies that enhance intrinsic motivation. For example, providing athletes with opportunities for all three dimensions of psychological needs within their sport can strengthen their intrinsic drive. Setting meaningful goals, offering constructive feedback, and fostering a positive team atmosphere could also boost motivation and build mental toughness.

In conclusion, this part emphasizes the key role of motivation in influencing college skiers’ experiences. Notably, autonomous motivation has a key role in enhancing athletes’ mental toughness, whereas a lack of motivation may negatively impact the development of mental toughness. These findings suggest the necessity for targeted interventions to cultivate autonomous motivation and address amotivation in order to promote athletes’ physical and mental health and improve their overall sports experience.

### 8.4. Practical Implications

The results have important practical implications for athletes’ well-being. By exploring the relationship between motivational factors and mental toughness in college skiers through the lens of the HMIEM, this study illuminates key motivational mechanisms that underpin mental toughness in athletes. Specifically, the results demonstrate that mental toughness is positively related to autonomous motivation and controlled motivation and negatively related to amotivation. These insights emphasize the critical role of coaching styles in fostering motivation and enhancing mental toughness during competition and training.

Identifying a series of motivational processes offers actionable guidance for coaches, trainers, and researchers. By understanding the role of motivation in cultivating mental toughness, practitioners can design tailored training programs, establish effective support systems, and promote a balanced, positive athletic experience. Such targeted interventions can not only improve mental toughness but also contribute to sustained athlete well-being and performance.

Additionally, this study enriches the broader understanding of the interplay between motivation and mental toughness, addressing a gap in research on skiers. The findings align with and extend the existing literature, reinforcing the theoretical foundation of the HMIEM. This expanded framework provides a valuable basis for investigating athlete well-being and performance from a more comprehensive perspective. Practitioners can build on these results to improve current models, develop novelty theories, and deepen their understanding of how motivation impacts mental toughness and overall mental health in athletes.

### 8.5. Limitations

This study has several limitations that warrant consideration. First, although the primary population comprised college skiers, the proportion of top-level skiers was limited, representing only 3.9% of the total sample. A larger sample of elite athletes would enable more robust and in-depth analyses. Second, the cross-sectional design limits its ability to examine temporal associations and dynamic processes, such as the evolving interplay between motivational factors and mental toughness over time. Considering evidence that mediation effects may vary across time (Maxwell & Cole, 2007), future research should adopt longitudinal designs to provide a more nuanced understanding of these relationships. Thirdly, the reliance on self-reported measures introduces the potential for mono-method bias, which may inflate correlations between variables for the observed effect sizes. Future studies should expand the scope to include additional dimensions of basic psychological needs, particularly the frustration of basic needs, or incorporate external observations of motivational climates from teammates or parents to mitigate this bias. Finally, since no further comparative analysis was conducted in the current research, we will focus on comparative research in future research, including sport type, gender, training volume, etc. By overcoming these limitations, future studies can build on the current findings to offer a deeper knowledge of the dynamic nature of motivation, mental toughness, and athletes’ well-being.

## 9. Conclusions

This study systematically examined the associations between mental toughness and motivation among college skiers in China, utilizing the HMIEM in a culturally specific context. While prior research has explored the link between motivation and mental toughness, this study makes a unique contribution by focusing on college skiers and addressing the challenges of incorporating mental toughness into the HMIEM framework. It provides a deeper understanding of how motivators work within a specific athlete population.

By investigating this relationship within a specific sport context—skiing—and employing a structural model to analyze the data, our findings provide critical insights that extend and enrich the existing literature on motivational processes and mental toughness in athletes. First, while the connection between motivation and mental toughness has been widely studied, these previous studies have often been conducted in broad, generalized contexts. By focusing on the relatively less explored domain of college skiers in China, this study offers a more nuanced perspective on how motivation influences mental toughness in a specific cultural and athletic environment.

Furthermore, this study’s use of the HMIEM adds a new dimension to our understanding of these motivational dynamics. The integration of mental toughness into this model, which incorporates the satisfaction of motivational climates, basic psychological needs, and sports motivation, allows for a more holistic examination of how motivation and mental toughness interact within the broader athlete experience. By demonstrating that specific motivational patterns may enhance mental toughness and, consequently, improve sports performance. This study extends the reach of the HMIEM model, providing researchers and practitioners with a comprehensive framework that can be applied to other sports contexts in the future. 

Ultimately, this study not only enhances the theoretical understanding of the relationship between motivation and mental toughness but also provides a rationale for improving athlete well-being and performance. By identifying key motivational factors that contribute to resilience and success in skiing, these findings offer actionable insights for developing training strategies that foster both psychological and athletic growth. The findings offer a foundation for future research exploring the motivational dynamics of other athlete populations, particularly those in culturally specific or underexplored contexts.

## Figures and Tables

**Table 1:** Demographic data of the participants.

Demographic	Male (*n* = 196) n (%)	Female (*n* = 136) n (%)	Total (*n* = 332) n (%)
Age			
18–19	53 (27.0)	33 (24.3)	86 (25.9)
20–21	80 (40.8)	53 (39.0)	133 (40.1)
22–23	41 (21.0)	32 (23.5)	73 (22.0)
24–25	22 (11.2)	18 (13.2)	40 (12.0)
Training time			
<5 years	21 (10.7)	30 (22.1)	51 (15.4)
5–7 years	86 (44.0)	66 (48.5)	152 (45.8)
8–10 years	77 (39.2)	21 (15.4)	98 (29.5)
>10 years	12 (6.1)	19 (14.0)	31 (9.3)
Training volume			
2–3 sessions/week	83 (42.3)	65 (47.8)	148 (44.6)
4–5 sessions/week	86 (43.9)	55 (40.4)	141 (42.5)
6 sessions/week	27 (13.8)	16 (11.8)	43 (12.9)
Sport items			
Cross-country skiing	25 (12.8)	11 (8.1)	36 (10.8)
Snowboarding	32 (16.3)	20 (14.7)	52 (15.7)
Freestyling	31 (15.8)	34 (25.0)	65 (19.6)
Alpine skiing	108 (55.1)	71 (52.2)	179 (53.9)
Competitive level			
National second level	82 (41.8)	72 (53.0)	154 (46.4)
National first level	90 (45.9)	41 (30.1)	131 (39.5)
National master level	19 (9.7)	15 (11.0)	34 (10.2)
International master level	5 (2.6)	8 (5.9)	13 (3.9)

**Table 2:** Descriptive statistics, reliability estimates, and bivariate correlations.

Variables	*M*/*SD*	*α*	Correlations										
			1	2	3	4	5	6	7	8	9	10	11
TC	4.31/0.70	0.93	-										
EC	3.08/0.98	0.91	−0.13 *	-									
COM	5.34/1.23	0.90	0.40 **	−0.21 **	-								
AUT	5.10/1.10	0.87	0.44 **	−0.30 **	0.78 **	-							
REL	5.82/1.11	0.81	0.45 **	−0.16 **	0.53 **	0.63 **	-						
AUTO MOTI	5.55/1.01	0.90	0.39 **	−0.27 **	0.41 **	0.46 **	0.45 **	-					
CTRL MOTI	3.74/1.24	0.83	0.02	0.03	0.16 **	0.12 *	0.10 *	0.26 **	-				
AMOTI	2.75/1.33	0.85	−0.12 **	0.18 **	−0.13 *	−0.20 **	−0.21 **	−0.15 **	0.20 **	-			
CONF	3.78/0.53	0.86	0.11 *	−0.11 **	0.15 *	0.18 **	0.18 **	0.42 **	0.21 **	−0.27 **	-		
CONS	3.75/0.56	0.85	0.10 *	−0.12 *	0.13 *	0.14 **	0.16 **	0.39 **	0.20 **	−0.25 **	0.88 **	-	
CONT	3.73/0.53	0.85	0.11 *	−0.12 *	0.11 *	0.14 **	0.14 **	0.38 **	0.15 **	−0.26 **	0.87 **	0.91 **	-

Note: TC = task-involving climate; EC = ego-involving climate; COM = competence; AUT = autonomy; REL = relatedness; AUTO MOTI = autonomous motivation; CTRL MOTI = controlled motivation; AMOTI = amotivation; CONF = confidence; CONS = constancy; CONT = control; * *p* < 0.05, ** *p* < 0.01.

**Table 3:** Standardized coefficients from the hypothesized model and the bootstrap analysis.

Path	Hypothesized Model	Bootstrap Analysis	Bias-Corrected 95% CI
	Standardized Coefficient	Mean Standardized Coefficient (SE)	
Task climate to psychological needs	0.47	0.47 (0.05)	0.38 to 0.57
Ego climate to psychological needs	−0.24	−0.25 (0.04)	−0.34 to −0.14
Psychological needs to autonomous motivation	0.53	0.53 (0.07)	0.41 to 0.63
Psychological needs to controlled motivation	0.15	0.15 (0.09)	0.25 to 0.38
Psychological needs to amotivation	−0.21	−0.22 (0.07)	−0.32 to −0.12
Autonomous motivation to mental toughness	0.33	0.33 (0.05)	0.17 to 0.48
Controlled motivation to mental toughness	0.15	0.15 (0.03)	0.05 to 0.25
Amotivation to mental toughness	−0.25	−0.25 (0.04)	−0.33 to −0.11

**Table 4:** Standardized indirect effects from the hypothesized model.

	Indirect Effect (*p* Value)	95% CI
TC–BPNS–AUTO MOTI–SMT	0.13 (0.01)	0.05 to 0.20
TC–BPNS–CTRL MOTI–SMT	0.06 (0.03)	0.01 to 0.12
TC–BPNS–AMOTI–SMT	0.07 (0.02)	0.01 to 0.13
EC–BPNS–AUTO MOTI–SMT	−0.05 (0.01)	−0.10 to −0.02
EC–BPNS–CTRL MOTI–SMT	−0.02 (0.07)	−0.06 to 0.00
EC–BPNS–AMOTI–SMT	−0.04 (0.02)	−0.07 to −0.02

Note: TC = task-involving climate; EC = ego-involving climate; BPNS = basic psychological needs satisfaction; AUTO MOTI = autonomous motivation; CTRL MOTI = controlled motivation; AMOTI = amotivation; SMT = sports mental toughness.

## Data Availability

Data cannot be shared openly but are available from the corresponding author upon reasonable request.

## Abbreviations

SDT: self-determination theory; AGT: achievement goal theory; HMIEM: hierarchical model of intrinsic and extrinsic motivation; SEM: structural equation modeling; PMCSQ-II: perceived motivational climate in sport questionnaire II; BNSSS: basic need satisfaction in sport scale; SMS-II: sport motivation scale II; SMTQ: sports mental toughness questionnaire; TC: task-involving climate; EC: ego-involving climate; BPNS: basic psychological needs satisfaction; COM: competence; AUT: autonomy; REL: relatedness; MOTI: motivation; SMT: sports mental toughness; CONF: confidence; CONS: constancy; CONT: control.
